# Implementing informative priors for heterogeneity in meta‐analysis using meta‐regression and pseudo data

**DOI:** 10.1002/sim.7090

**Published:** 2016-08-30

**Authors:** Kirsty M. Rhodes, Rebecca M. Turner, Ian R. White, Dan Jackson, David J. Spiegelhalter, Julian P. T. Higgins

**Affiliations:** ^1^ MRC Biostatistics UnitCambridge Institute of Public HealthCambridgeU.K.; ^2^ Statistical LaboratoryCentre for Mathematical Sciences, University of CambridgeU.K.; ^3^ School of Social and Community MedicineUniversity of BristolU.K.

**Keywords:** meta‐analysis, heterogeneity, informative priors, meta‐regression, data augmentation

## Abstract

Many meta‐analyses combine results from only a small number of studies, a situation in which the between‐study variance is imprecisely estimated when standard methods are applied. Bayesian meta‐analysis allows incorporation of external evidence on heterogeneity, providing the potential for more robust inference on the effect size of interest. We present a method for performing Bayesian meta‐analysis using data augmentation, in which we represent an informative conjugate prior for between‐study variance by pseudo data and use meta‐regression for estimation. To assist in this, we derive predictive inverse‐gamma distributions for the between‐study variance expected in future meta‐analyses. These may serve as priors for heterogeneity in new meta‐analyses. In a simulation study, we compare approximate Bayesian methods using meta‐regression and pseudo data against fully Bayesian approaches based on importance sampling techniques and Markov chain Monte Carlo (MCMC). We compare the frequentist properties of these Bayesian methods with those of the commonly used frequentist DerSimonian and Laird procedure. The method is implemented in standard statistical software and provides a less complex alternative to standard MCMC approaches. An importance sampling approach produces almost identical results to standard MCMC approaches, and results obtained through meta‐regression and pseudo data are very similar. On average, data augmentation provides closer results to MCMC, if implemented using restricted maximum likelihood estimation rather than DerSimonian and Laird or maximum likelihood estimation. The methods are applied to real datasets, and an extension to network meta‐analysis is described. The proposed method facilitates Bayesian meta‐analysis in a way that is accessible to applied researchers. © 2016 The Authors. *Statistics in Medicine* Published by John Wiley & Sons Ltd.

## 1 Introduction

In a meta‐analysis, results of similar studies are combined in order to synthesise evidence available in a specific research area. Differences among the results of the included studies arise through differences in study design, through biases because of methodological flaws and through random variation. When between‐study heterogeneity exists, it may be appropriate to fit a random‐effects meta‐analysis model, estimating a between‐study variance and a summary effect 1. Conventional methods to estimate the random‐effects meta‐analysis model are justifiable provided that the number of studies is sufficiently large. However, numerous meta‐analyses in healthcare research combine results from only a small number of studies: of 22 453 meta‐analyses within the *Cochrane Database of Systematic Reviews* (*CDSR*), approximately 75*%* contained five or fewer studies 2. For such meta‐analyses, between‐study variance is imprecisely estimated when conventional methods are applied.

A Bayesian approach to random‐effects meta‐analysis is beneficial in allowing a researcher to incorporate evidence on the likely extent of heterogeneity, providing the potential for more robust inference on the summary effect of interest 3, 4, 5, 6. Other advantages of Bayesian meta‐analysis include full allowance for all sources of parameter uncertainty, the opportunity to ‘ borrow strength’ from other studies in estimating an individual effect, and the ability to make predictions about the true intervention effects of future studies 1. Bayesian methods offer a flexibility, which allows us to perform more complex analyses. Despite the advantages of Bayesian methods, the vast majority of published meta‐analyses are frequentist. Recently, however, Turner *et al.* 7 have proposed two easy‐to‐use methods for implementing fully Bayesian meta‐analyses using importance sampling techniques and numerical integration. The methods do not have problems with convergence or mixing as in Markov chain Monte Carlo (MCMC), and they have given similar meta‐analysis results in example applications 7.

The standard approach for conducting Bayesian meta‐analysis is to use simulation based methods such MCMC methods within the *WinBUGS* (MRC Biostatistics unit, Cambridge, U.K.) software 8. Although MCMC methods are powerful for solving complex problems, they are computationally intensive and can be potentially misleading if used without care 9. In a typical meta‐analysis containing data from only a small number of studies, computational efficiency is not an issue. However, meta‐analyses are very often performed by applied researchers with only basic statistical training, who may be unfamiliar with the implementation of MCMC methods. The use of MCMC methods requires the researcher to be confident in determining whether or not the MCMC results may be considered valid to report.

A simpler method for Bayesian analysis is data augmentation 10, 11, 12, 13, 14, in which a conjugate prior distribution is represented by pseudo data. Data augmentation may be considered to provide a semi‐Bayesian analysis, because it does not require prior distributions to be specified for unknown parameters. The approach allows one to implement approximate Bayesian analyses using commonly used frequentist methods within standard statistical regression software, thus removing the need to rely on specialist software. It requires no simulation at all, and therefore runs much faster than methods using MCMC and importance sampling techniques.

All Bayesian meta‐analyses require prior distributions to be declared for unknown parameters. A prior is referred to as a conjugate prior if the resulting posterior distribution is of the same parametric family as the prior. In Bayesian analysis, use of a conjugate prior is sometimes preferred because the prior is computationally and mathematically easy to deal with. An additional advantage of using a conjugate prior is that the prior is interpretable as additional data 15, which will be useful in the data augmentation method we develop in this paper. To enable a systematic reviewer to implement Bayesian meta‐analyses by data augmentation, it is desirable for conjugate prior distributions, describing the expected magnitude of between‐study variance *τ* ^2^ in different research settings, to be made available. Researchers carrying out a meta‐analysis could then select the informative prior distribution most suitable for the characteristics of their meta‐analysis.

The main contribution of this paper is to show that methods for Bayesian analysis by data augmentation may be extended to Bayesian random‐effects meta‐analysis with an informative prior for the between‐study variance. A new set of predictive distributions for the between‐study variance expected in binary outcome meta‐analyses is reported, to facilitate Bayesian meta‐analysis with an informative conjugate inverse‐gamma prior for between‐study variance rather than existing log‐normal priors 5. This paper formally compares approximate Bayesian methods using data augmentation against fully Bayesian approaches using MCMC methods and importance sampling techniques, with computing code for all methods available in Supporting Information. The frequentist properties of the Bayesian methods are compared with those of the commonly used DerSimonian and Laird (DL) procedure. We demonstrate that informative priors for between‐study variance derived for meta‐analysis can also be used in network meta‐analysis. As an example, we use a simple network meta‐analysis comparing the effects of four different interventions for smoking cessation counselling.

The rest of the paper is set out as follows. In Section 2, we present our method for implementing a conjugate prior for the between‐study variance in an approximate Bayesian meta‐analysis. In order to provide users with informative priors of the form developed in Section 2, Section 3 derives a new set of predictive inverse‐gamma distributions for between‐study variance expected in future binary outcome meta‐analyses. We then apply our proposed method to two contrasting examples in Section 4 and to simulation studies in Section 5, incorporating the predictive distributions obtained in Section 3 as prior distributions for between‐study heterogeneity. In Section 6, we apply our method to a simple network meta‐analysis. We conclude with a discussion in Section 7.

## 2 Methods

In this section, we develop a method for implementing Bayesian meta‐analysis based on data augmentation and describe the standard approach using MCMC methods. The random‐effects model 16, 17, 18 frequently used in meta‐analysis assumes that

$$Y_{i}∼N\left(\mu ,\sigma_{i}^{2}+\tau^{2}\right),$$

where *Y*_*i*_ is the observed intervention effect in study *i* (*i* = 1,2,…,*K*), *μ* is the summary intervention effect for the meta‐analysis and *τ* ^2^ is the between‐study variance. The within‐study variances $\sigma_{i}^{2}$ are estimated in practice but are treated as fixed and known in the analysis.

### 2.1 Data augmentation

This research aims to develop a method for implementing a conjugate prior for *τ* ^2^ in a meta‐analysis, using data augmentation. The basic idea is that a conjugate prior can be expressed as additional data. This leads to a simple process for conducting an approximate Bayesian meta‐analysis within standard statistical software:

1. Construct pseudo data ‘equivalent’ to the prior;
2. Add those pseudo data to the observed study data as a distinct stratum;
3. Fit a random‐effects meta‐regression model.

As a single covariate in the meta‐regression model, we include an indicator for whether the data are observed. The resulting estimates from the frequentist analysis of the augmented (pseudo+observed) data correspond to approximate posterior estimates for each parameter.

#### 2.1.1 Meta‐regression model and pseudo data

In a general random‐effects meta‐regression model, we assume

$$Y_{i}|x_{i}∼N\left(x_{i}\beta ,\sigma_{i}^{2}+\tau^{2}\right),$$

where *Y*_*i*_ is the estimated effect from the *i*‐th study (*i* = 1,2,…,*K*), *x*_*i*_ is the 1 × *p* vector of covariates associated with this study and ***β*** is the vector of regression coefficients. Typically, the first ‘covariate’ is taken to be one to include an intercept, unless an intercept free regression is required.

Our proposed approach to implement a prior for the between‐study variance *τ* ^2^ in a meta‐analysis is to augment the observed data from *K* studies using pseudo data from *K*_0_ artificial studies. We propose the following random‐effects meta‐regression for both the observed and pseudo data:

(1) $$Y_{i}|x_{i}∼N\left(x_{i}\mu ,\sigma_{i}^{2}+\tau^{2}\right),$$

where *Y*_*i*_ is the intervention effect from the *i*‐th study (*i* = 1,…,*K* + *K*_0_), and the covariate *x*_*i*_ in each study is taken to be one if the study is observed or zero if the study is artificial (i.e. representing the prior for *τ* ^2^). We set the intercept term to zero so that the effects of the artificial studies are centred at zero, and *μ* estimates the combined effect of the *K* observed studies. The residual between‐study heterogeneity *τ* ^2^ is not modified by the indicator *x*_*i*_ (we assume the same residual between‐study variance for the observed and pseudo data), and so, between‐study variation is incorporated in the same way as in a standard random‐effects meta‐analysis.

In other words, the observed data are modelled by

$$Y_{i}∼N\left(\mu ,\sigma_{i}^{2}+\tau^{2}\right),\;i=1,\ldots ,K,$$

and so the pseudo data from the artificial studies are modelled by

$$Y_{i}∼N(0,\tau^{2}),\;i=K+1,\ldots ,K+K_{0}.$$

It is mathematically convenient and standard practice in Bayesian analysis to work in terms of precision. The likelihood of the between‐study precision *ϕ* = 1/*τ* ^2^ from the artificial studies $y_{K+1},...y_{K+K_{0}}$ is

$$L(\phi )=\overset{K+K_{0}}{\underset{i=K+1}{\prod }}p(y_{i}|\phi )\propto \phi^{K_{0}/2}\exp\left\{-\frac{\phi }{2}\overset{K+K_{0}}{\underset{i=K+1}{\sum }}y_{i}^{2}\right\}.$$

Creation of the data for augmenting requires us to generate an effect *y*_*i*_ for each artificial study indexed *i*(*i* = *K* + 1,…,*K* + *K*_0_). Only $\sum_{i=K+1}^{K+K_{0}}y_{i}^{2}$ matters (see previous equation), and so, we may as well choose the simplest case of homogeneous artificial studies, all with effect *y*_0_. Then, the likelihood contribution from the artificial studies $y_{K+1},...y_{K+K_{0}}$ is

(2) $$L(\phi )=\prod_{i=K+1}^{K+K_{0}}p(y_{i}|\phi )\propto \phi^{K_{0}/2}\exp\left\{-\frac{\phi }{2}K_{0}y_{0}^{2}\right\}.$$

Gamma priors are commonly assigned to precision parameters in Bayesian analyses. The intuition behind our proposed method to implement an inverse‐gamma prior for *τ* ^2^, and so a gamma prior for *ϕ*, is that the aforementioned likelihood for *ϕ* = 1/*τ* ^2^ is proportional to the following probability density function of a Gamma (*α*,*β*) distribution:

$$p(\phi )=\frac{\beta^{\alpha }}{\Gamma (\alpha )}\phi^{\alpha -1}\exp(-\beta \phi ),$$

where *α* = *K*_0_/2 + 1 and $\beta =\frac{1}{2}K_{0}y_{0}^{2}$.

Suppose that we want to implement a generic inverse‐gamma(*α*,*β*) prior for *τ* ^2^ in a Bayesian meta‐analysis. In order to implement this prior using data augmentation, we need to match the likelihood of the between‐study precision *ϕ* = 1/*τ* ^2^ given the data *y*_*i*_(*i* = *K* + 1,…,*K* + *K*_0_) from the artificial studies to the Gamma(*α*,*β*) prior density. To replace a prior density with a likelihood term, the prior density needs to be equivalent to a posterior given by the likelihood multiplied by a uniform prior. We therefore need a parameter on the whole real line for which a flat prior is appropriate, and so, we work with the logarithm of the between‐study precision *φ* = log*ϕ* =− 2log*τ*‐ this re‐parametrisation will not affect the maximum‐likelihood analysis for which we are aiming. Based on a standard result for transforming random variables 19, the Gamma(*α*,*β*) prior density for the between‐study precision *ϕ* in terms of its logarithm *φ* = log*ϕ* is, upon changing variable in the usual way,

$$\begin{array}{cc} p_{\varphi }(\varphi ) & =p_{\phi }(\exp(\varphi ))|\exp(\varphi )|\\ \propto \exp((\alpha -1)\varphi )\exp(-\beta \exp(\varphi ))|\exp(\varphi )|\\ \propto \exp(\alpha \varphi -\beta \exp(\varphi )), \end{array}$$

where | exp(*φ*)| is the Jacobian of the transformation.

In terms of the logarithm of the between‐study precision *φ*, the likelihood contribution from the pseudo data in equation ((2)) is

$$L(\varphi )\propto \exp\left(\frac{K_{0}}{2}\varphi -\frac{K_{0}y_{0}^{2}}{2}\exp\varphi \right).$$

This likelihood will exactly match the contribution for the prior density for *φ* if we choose *K*_0_ and *y*_0_ such that *α* = *K*_0_/2 and $\beta =K_{0}y_{0}^{2}/2$. That is, we set *K*_0_ = 2*α* and $y_{0}=\sqrt{2\beta /K_{0}}$.

The intervention effect *y*_0_ chosen for the artificial studies and the number of artificial studies *K*_0_ therefore govern the prior mean on *τ* ^2^; an inverse‐gamma(*α*,*β*) prior has mean *β*/(*α* − 1) that increases with $\beta =K_{0}y_{0}^{2}/2$. The number of artificial studies *K*_0_ determines the desired prior precision; the variance of the inverse‐gamma(*α*,*β*) prior is *β* ^2^/((*α* − 1)^2^(*α* − 2)), which decreases as *α* = *K*_0_/2 increases. Visual representations of pseudo data to represent inverse‐gamma prior distributions for the between‐study variance *τ* ^2^ are given in the Supporting Information (S1).

#### 2.1.2 Proposed method

In summary, our proposed approach for performing an approximate Bayesian random‐effects meta‐analysis with an inverse‐gamma(*α*,*β*) prior for the between‐study variance *τ* ^2^ is to

1. Add *K*_0_ = 2*α* precise studies (rounded to the nearest integer) with intervention effects $y_{0}=\sqrt{2\beta /K_{0}}$;
2. Model these as having mean zero, the same heterogeneity *τ* ^2^ as the observed data, and within‐study variances $\sigma_{i}^{2}$ of approximately zero (we set each $\sigma_{i}^{2}=10^{-20}$);
3. Implement using a standard method to estimate the random‐effects meta‐regression model.

Note that our approach of adding extra studies to represent a prior for the between‐study variance *τ* ^2^ in a meta‐analysis does not introduce bias in the intervention effect because we set the mean of the artificial studies to zero so that they do not contribute directly to the point estimate for the summary intervention effect *μ*. The extra studies do, however, affect the point estimate for the between‐study variance *τ* ^2^ and the mean squared error (MSE).

*R* and *Stata* code to perform an approximate Bayesian meta‐analysis using meta‐regression and pseudo data is given in the Supporting Information (S2.1).

Conceptually, we replace the prior distribution for *τ* ^2^ with an equivalent likelihood contribution using the pseudo data.

### 2.2 Methods to estimate the meta‐regression model

Our meta‐regression involves two parameters: the mean *μ* and the variance *τ* ^2^ of the random‐effects distribution. The between‐study variance *τ* ^2^ is usually estimated and then treated as fixed and known when drawing inference about the intervention effect 17. This approximation performs well in large samples. The most commonly used method to estimate this variance is the method‐of‐moments estimator proposed by DL 16. Maximum likelihood (ML) and restricted maximum likelihood (REML) estimation are alternative procedures that require iteration and are therefore more computationally intensive. In this paper, we investigate the use of these three methods for implementing an approximate Bayesian meta‐analysis using meta‐regression and pseudo data.

### 2.3 Markov chain Monte Carlo methods

Markov chain Monte Carlo methods can be used to obtain summary statistics for the joint posterior distribution of *μ* and *τ* ^2^, within the *WinBUGS* 8 software. We based results on 1 000 000 iterations, following a burn‐in period of 10 000 iterations, which was sufficient to achieve convergence and produced very low MC error rates. Convergence was assessed using the Brooks–Gelman–Rubin statistic 20, with five chains starting from widely dispersed initial values, as implemented in *WinBUGS*. We declared a vague *N*(0,10^6^) prior for the summary intervention effect *μ*. *WinBUGS* code to perform Bayesian random‐effects meta‐analysis by MCMC is given in the Supporting Information (S2.2).

## 3 Informative priors for heterogeneity

We now have some elegant theory for conducting an approximate Bayesian meta‐analysis with an inverse‐gamma prior for *τ* ^2^. This was developed by working with the logarithm of the between‐study precision 1/*τ* ^2^ and matching the corresponding likelihood from some pseudo data to the Gamma prior for 1/*τ* ^2^ in terms of  − 2log(*τ*). To use our method described in Section 2.1.2, we need inverse‐gamma prior distributions for the between‐study variance *τ* ^2^.

Previously, we have provided ‘off‐the‐shelf’ data‐based prior distributions for the between‐study variance *τ* ^2^ in a meta‐analysis 5, 6. Turner *et al.* 5 and Rhodes *et al.* 6 investigated the influence of meta‐analysis characteristics on between‐study heterogeneity in a meta‐analysis. Turner *et al.* obtained predictive log‐normal distributions for *τ* ^2^ expected in future binary outcome meta‐analyses of log odds ratios in each of nine different research settings. Rhodes *et al.* reported predictive log‐*t*_5_ and inverse‐gamma distributions for *τ* ^2^ expected in future continuous outcome meta‐analyses of standardised mean differences. The problem in using the existing predictive distributions available for *τ* ^2^ in binary outcome meta‐analyses as priors in our new data augmentation method is that they are log‐normal and cannot be represented as pseudo data in the same way as conjugate prior distributions. In the absence of informative inverse‐gamma prior distributions, one possibility would be to match the mean and variance of the published log‐normal prior distributions to provide inverse‐gamma prior distributions. However, this is far from ideal because the mean and variance alone cannot describe the inverse‐gamma distribution well as this distribution is skew. A better approach is to construct inverse‐gamma prior distributions using a suitable database of meta‐analyses, and we derive prior distributions of this type below. Our approach is to repeat the procedures previously used for constructing informative priors for between‐study heterogeneity *τ* ^2^, in this case, assuming an inverse‐gamma distribution for underlying values of between‐study heterogeneity rather than a log‐normal or log‐*t*_5_ prior distribution.

### 3.1 Data set

The Nordic Cochrane centre provided us with the study data from meta‐analyses included in the *CDSR* (Issue 1, 2008). Most Cochrane reviews include multiple meta‐analyses, which correspond to comparisons of interventions and the assessment of various outcomes within these comparisons. In some reviews, results from studies eligible for a meta‐analysis were available but no combined results were present. We treated these data in the same way as meta‐analyses, because the extent of heterogeneity may have influenced the decision not to report the pooled meta‐analysis results. Reviews sometimes present results for several subgroup analyses within meta‐analyses. Because we are interested in the overall between‐study heterogeneity in a meta‐analysis, study results were combined across subgroups. In some reviews, the subgroups presented within a meta‐analysis were not mutually exclusive; therefore, we checked for study duplications and used data for only the first occurrence of each study in each meta‐analysis 2.

For computational convenience, we analysed data from the first reported meta‐analysis per pair‐wise intervention comparison, which included at least two studies. In total, the data set analysed includes 3873 binary outcome meta‐analyses from 1967 Cochrane reviews, containing data from 21 902 individual studies. This same data set has been analysed previously for the purpose of obtaining predictive distributions for the *I* ^2^ measure 21. Each meta‐analysis in the database was classified according to the type of outcome, types of intervention compared and medical speciality, as described in an earlier paper 2. The outcomes, interventions and medical specialities were assigned to fairly narrow categories, which we grouped together in the same way as Turner *et al.* 5. Outcomes were categorised into groupings of ‘all‐cause mortality’, ‘semi‐objective’ and ‘subjective’. ‘Semi‐objective’ outcomes are those that are objectively measured but potentially influenced by judgement, for example caesarean section, hospital admission and study withdrawal. ‘Subjective’ outcomes include self‐reported outcomes such as pain and satisfaction with care, and outcomes measured by an assessor whose method of measurement and judgement may influence the outcome, for example hypertension and infection. The distributions of outcome types, intervention comparison types and medical areas are summarised elsewhere in 21. The Cochrane database covers a very wide range of medical areas. ‘Obstetrics and gynaecology’ was the most frequent medical area (18*%* of meta‐analyses), followed by ‘Mental health and behavioural conditions’ (14*%* of meta‐analyses).

### 3.2 Inverse‐gamma prior distributions obtained

We used Bayesian hierarchical models to analyse study data from each binary outcome meta‐analysis in the data set, while investigating the influence of meta‐analysis characteristics on the degree of heterogeneity among results of included studies. For each research setting defined by the type of outcome and type of intervention comparison, we obtained a predictive distribution for heterogeneity $\tau_{new}^{2}$ in a new meta‐analysis in that setting, within the full Bayesian model. Details of the hierarchical models for heterogeneity are available in the Supporting Information (S3) and have been described elsewhere in 5, 6. We first report a predictive distribution for the between‐study variance expected in a future meta‐analysis in a generic health‐care setting. This was obtained from a hierarchical model fitted to all 3873 meta‐analyses in the data set, without including covariates representing meta‐analysis characteristics. From this model, the fitted distribution for $\tau_{new}^{2}$ is estimated as inverse‐gamma(1.14,0.08), which has median 0.09 and 95*%* range (0.02, 1.79).

Table 1 summarises a set of inverse‐gamma distributions fitted to the predictive distributions for between‐study variance expected in a future meta‐analysis in each of nine different settings, defined by the type of outcome and the type of intervention comparison. The differences among the different fitted distributions reflect the findings reported by Turner *et al.* 5. There are clear differences across the different outcome types; the predictive distributions for meta‐analyses of an all‐cause mortality outcome have much lower quantiles, whereas the fitted distributions for a subjective outcome have the highest quantiles. Within outcome types, discrepancies among the different types of intervention comparisons seem small, but levels of heterogeneity tend to be lower in pharmacological versus pharmacological comparisons.

**Table 1:** Predictive inverse‐gamma (IG) distributions for between‐study variance *τ* ^2^ expected in future binary outcome meta‐analyses of log odds ratios. To implement a predictive distribution as an informative prior for *τ* ^2^ in an approximate Bayesian meta‐analysis by data augmentation, we augment the observed study data using the pseudo data reported.

	**Pharmacological versus**	**Pharmacological versus**	**Non‐pharmacological**
	**placebo/ control**	**pharmacological**	**(any)**
	*I* *G*(1.06,0.01)	*I* *G*(2.93,0.00003)	*I* *G*(0.80,0.007)
**All‐cause mortality**	**Pseudo data:**	**Pseudo data:**	**Pseudo data:**
	2 studies with	6 studies with	2 studies with
	effects *y*_0_ = 0.100	effects *y*_0_ = 0.003	effects *y*_0_ = 0.084
	*I* *G*(1.32,0.08)	*I* *G*(1.04,0.04)	*I* *G*(0.88,0.05)
**Semi‐objective**	**Pseudo data:**	**Pseudo data:**	**Pseudo data:**
	3 studies with	2 studies with	2 studies with
	effects *y*_0_ = 0.231	effects *y*_0_ = 0.200	effects *y*_0_ = 0.224
	*I* *G*(1.45,0.18)	*I* *G*(1.13,0.09)	*I* *G*(1.39,0.13)
**Subjective**	**Pseudo data:**	**Pseudo data:**	**Pseudo data:**
	3 studies with	2 studies with	3 studies with
	effects *y*_0_ = 0.346	effects *y*_0_ = 0.300	effects *y*_0_ = 0.294

For each predictive distribution reported in Table 1, we provide the pseudo data that should be used to implement the predictive distribution as an informative prior for *τ* ^2^ in an approximate Bayesian meta‐analysis by data augmentation. The intervention effects *y*_0_ of the pseudo study data are highest in magnitude for subjective outcome meta‐analyses and lowest in magnitude for meta‐analyses assessing all‐cause mortality. Across the three different types of intervention comparisons, the intervention effects *y*_0_ of the pseudo study data are consistently lower in magnitude for pharmacological versus pharmacological comparisons.

## 4 Applications to example meta‐analyses

In this section, we apply five different methods to two contrasting examples.

### 4.1 Example 1: fluoride for lower limb pain in patients with postmenopausal osteoporosis

The first example is a binary outcome meta‐analysis combining results from four studies evaluating the effectiveness of fluoride for lower limb pain in patients with postmenopausal osteoporosis 22. In a conventional random‐effects meta‐analysis by DL estimation 16, the estimate of between‐study heterogeneity is high at 1.78, and imprecisely estimated (95*%* CI: 0.39 to 52.2 obtained iteratively using the Q‐profile method 23). The summary log odds ratio is estimated as 1.42 (95*%* CI: ‐0.08 to 2.91). This meta‐analysis compares a pharmacological intervention against a control, with respect to a subjective outcome, so we choose an inverse‐gamma(1.45, 0.18) distribution as an informative prior for *τ* ^2^ (Table 1), which has a median of 0.16, and 95*%* range 0.04 to 1.87.

When implementing an inverse‐gamma(1.45, 0.18) prior distribution for *τ* ^2^ using MCMC methods, the central estimate (posterior median) for *τ* ^2^ reduces to 0.52, with 95*%* credible interval 0.07 to 4.21 (Table 2). In the Bayesian meta‐analysis, the combined log odds ratio for the intervention effect reduces to 1.24 (95*%* CI: 0.18 to 2.56).

**Table 2:** Results from re‐analysing data from published meta‐analyses to explore discrepancies between data augmentation and MCMC.

		**Prior assigned**	**Achieved prior**					
**Method**	**Data set**	**to** *τ* ^2^	**for** *τ* ^2^	**Log OR** (95*%* **CI**)	**MC error**	*τ* ^2^ (95*%* **CI**)	**MC error**	*I* ^2^
**Review: Fluoride for treating postmenopausal osteoporosis**								
**Comparison: Fluoride vs control. Outcome: lower limb pain**								
Conventional (DL estimation)	Observed			1.42( − 0.08,2.91)		1.78(0.39,52.2)^1^		80*%*
Data augmentation by DL	Augmented^4^	IG(1.45, 0.18)^3^	IG(1.5, 0.18)	1.15(0.48,1.83)		0.12(0.17,8.48)^1^		
Data augmentation by ML	Augmented^4^	IG(1.45, 0.18)^3^	IG(1.5, 0.18)	1.21(0.35,2.07)		0.36(0.17,8.48)^1^		
Data augmentation by REML	Augmented^4^	IG(1.45, 0.18)^3^	IG(1.5, 0.18)	1.24(0.30,2.19)		0.51(0.17,8.48)^1^		
MCMC	Observed	IG(1.45, 0.18)^3^	IG(1.45, 0.18)	1.24(0.18,2.56)^2^	0.0009	0.52(0.07,4.21)^2^	0.003	
**Results for exploring discrepancies between data augmentation and MCMC**								
MCMC	Augmented^4^	*τ* ∼ IG(0.001, 0.001)	IG(1.5, 0.18)	1.24(0.20,2.53)^2^	0.0006	0.50(0.07,3.97)^2^	0.002	
MCMC	Observed	IG(1.5, 0.18)^5^	IG(1.5, 0.18)	1.24(0.20,2.53)^2^	0.0006	0.50(0.07,3.98)^2^	0.002	
**Review: Antibiotics for preterm rupture of membranes**								
**Comparison: Penicillin (excluding co‐amoxiclav) vs placebo. Outcome: death before discharge**								
Conventional (DL estimation)	Observed			− 0.28( − 1.28,0.72)		0(0,7.61)^1^		0*%*
Data augmentation by DL	Augmented^4^	IG(1.06, 0.01)^6^	IG(1, 0.01)	− 0.28( − 1.29,0.73)		0.01(0.002,0.52)^1^		
Data augmentation by ML	Augmented^4^	IG(1.06, 0.01)^6^	IG(1, 0.01)	− 0.28( − 1.29,0.73)		0.01(0.002,0.52)^1^		
Data augmentation by REML	Augmented^4^	IG(1.06, 0.01)^6^	IG(1, 0.01)	− 0.28( − 1.29,0.73)		0.01(0.002,0.52)^1^		
MCMC	Observed	IG(1.06, 0.01)^6^	IG(1.06, 0.01)	− 0.28( − 1.31,0.74)^2^	0.003	0.01 (0.003,0.21)^2^	0.0002	

**Results for exploring discrepancies between data augmentation and MCMC**								
MCMC	Augmented^4^	*τ* ∼ IG(0.001, 0.001)	IG(1, 0.01)	− 0.28( − 1.31,0.74)^2^	0.003	0.01(0.003,0.24)^2^	0.0002	
MCMC	Observed	IG(1, 0.01)^5^	IG(1, 0.01)	− 0.28( − 1.31,0.74)^2^	0.003	0.01 (0.003,0.24)^2^	0.0002	

1 The confidence interval for *τ* ^2^ is obtained iteratively via the Q‐profile method 23.

2 Posterior medians and 95*%* credible intervals are reported.

3 Empirically based inverse‐gamma(1.45,0.18) prior for a pharmacological vs placebo/control meta‐analysis with a subjective outcome.

4 Observed data augmented using pseudo data to represent the achieved inverse‐gamma prior for *τ* ^2^.

5 The prior we achieve in data augmentation, due to rounding the number of artificial studies to be an integer.

6 Empirically based inverse‐gamma(1.06,0.01) prior for a pharmacological vs placebo/control meta‐analysis with an all‐cause mortality outcome.

MCMC, Markov chain Monte Carlo; DL, DerSimonian and Laird; ML, maximum likelihood; REML, restricted maximum likelihood.

### 4.2 Example 2: penicillin for prevention of perinatal death/ death prior to discharge from hospital

As a second contrasting example, we also re‐analyse data from a published meta‐analysis in which heterogeneity is low, but again imprecisely estimated (*τ* ^2^ = 0, 95*%* CI: 0 to 7.61). This meta‐analysis was taken from a Cochrane review with the objective to assess whether certain antibiotics given to women whose waters have broken early will improve their babies health. The meta‐analysis compares all penicillin (excluding co‐amoxiclav) against a placebo in terms of perinatal death/death prior to discharge from hospital 24. When using MCMC to perform Bayesian meta‐analysis with an informative inverse‐gamma(1.06, 0.01) prior for *τ* ^2^, the central estimate for *τ* ^2^ increases slightly to 0.01, and the 95*%* credible interval narrows considerably to (0.003, 0.21). The Bayesian approach produces a slightly wider interval for the combined log odds ratio in comparison with a conventional random‐effects meta‐analysis by DL estimation (Table 2), allowing for uncertainty in *τ* ^2^.

### 4.3 Exploring discrepancies between data augmentation and Markov chain Monte Carlo

In both examples of applying an informative inverse‐gamma prior distribution for *τ* ^2^, the point estimate for the effect size of interest obtained using the DL procedure by data augmentation is close to that resulting from the standard approach by MCMC (Table 2). However, the approach yields a noticeably smaller estimate for the between‐study variance in Example 1. For the first example meta‐analysis in which heterogeneity is high, point estimates for both the combined log odds ratio and *τ* ^2^ are closer to those from MCMC, if data augmentation is implemented using ML estimation instead of the DL procedure.

A standard method to estimate the meta‐regression model is to use REML estimation. Therefore, we explore how results from data augmentation would compare to MCMC, if REML estimation is used instead of DL or ML estimation. For the first example meta‐analysis in which heterogeneity is high, point estimates for both the combined log odds ratio and *τ* ^2^ are closer to those from MCMC, if data augmentation is implemented using REML estimation. For the second example in which heterogeneity is low, results obtained through data augmentation are almost identical, regardless of the method of implementation. In both examples, the interval for *τ* ^2^ resulting from data augmentation is wider than the interval resulting from MCMC. This is as expected because the Q‐profile method is not fully efficient for ML estimators. We note that the estimate of *τ* ^2^ in data augmentation is more precise than in the conventional meta‐analysis using the DL procedure.

The results obtained through data augmentation are similar to those from MCMC but show some discrepancies (Table 2). We would not expect data augmentation to lead to precisely the same results as MCMC because data augmentation provides an approximate Bayesian analysis, and MCMC is affected by simulation error. The extent to which these discrepancies are due to the simulation error in MCMC or the rounding of the prior shape parameter in data augmentation is investigated in this section. For this investigation, we again apply MCMC and data augmentation methods to study data from the two example meta‐analyses. In each example, we consider two different prior distributions for *τ* ^2^: the empirically based inverse‐gamma(*α*,*β*) prior and the inverse‐gamma prior with the shape parameter *α* rounded to force the number of artificial studies *K*_0_ = 2*α* to be an integer. For the first meta‐analysis, we chose to implement an empirically based inverse‐gamma(1.45,0.18) prior for *τ* ^2^ and an inverse‐gamma(1.5,0.18) prior for *τ* ^2^. For the second meta‐analysis, we chose to implement an empirically based inverse‐gamma(1.06,0.01) prior for *τ* ^2^ and an inverse‐gamma(1,0.01) prior for *τ* ^2^.

When implementing an inverse‐gamma(*α*,*β*) prior such that *K*_0_ = 2*α* is an integer, differences between point estimates obtained through MCMC and data augmentation are not mathematical but arise due to the fact that data augmentation is not a fully Bayesian approach. In data augmentation, we do not need to specify priors for all unknown parameters, therefore not all sources of parameter uncertainty are accounted for. In both examples, the implementation of an inverse‐gamma(*α*,*β*) prior constructed to force 2*α* to be an integer by MCMC leads to a very similar estimate for the intervention effect size of interest to that obtained through data augmentation methods (Table 2). Point estimates for the between‐study variance *τ* ^2^ resulting from data augmentation and MCMC show more noticeable discrepancies. Again, we find that point estimates for model parameters are consistently closer to those based on MCMC methods, when data augmentation is implemented using REML estimation instead of ML estimation.

For each of the two examples, we also analyse the augmented data set used for meta‐analysis by data augmentation, under a fully Bayesian framework, using MCMC within *WinBUGS*. For these analyses, we declare a vague normal(0,10^6^) prior for unknown location parameters and an inverse‐gamma(0.001, 0.001) prior for *τ*. When implementing an inverse‐gamma(*α*,*β*) prior constructed such that 2*α* is an integer, the results from analysing only the augmented data set using MCMC with a vague prior for heterogeneity are almost identical to those from analysing only the observed data using MCMC with the same inverse‐gamma(*α*,*β*) prior for heterogeneity (Table 2). The comparability of MCMC results indicates that the pseudo data we use to augment the observed study data in data augmentation are appropriate.

When implementing an empirically based inverse‐gamma prior for *τ* ^2^, the MCMC results based on analyses of the observed data alone with an informative prior for heterogeneity are quite similar to those based on analyses of the augmented data set with a vague prior for heterogeneity (Table 2). However, in view of the stronger similarities between these results when implementing an inverse‐gamma(*α*,*β*) prior for *τ* ^2^ constructed such that 2*α* is an integer, it is clear that differences between MCMC results and those obtained using data augmentation may arise because of the rounding of prior parameters in an empirically‐based Bayesian meta‐analysis by data augmentation.

Meta‐analysts should be aware that our data augmentation method for implementing Bayesian meta‐analysis will achieve a slightly different prior for the between‐study variance *τ* ^2^ to the inverse‐gamma(*α*,*β*) prior declared, if 2*α* is non‐integer. When 2*α* is an integer, the results in this section suggest that the pseudo data constructed to represent the inverse‐gamma(*α*,*β*) prior are appropriate.

## 5 A simulation study

We conducted a simulation study with three objectives: (i) to compare the frequentist properties of the Bayesian methods with those of the DL approach; (ii) to compare approximate Bayesian methods using data augmentation against a fully Bayesian approach using importance sampling techniques; and (iii) to show that importance sampling techniques yield approximately the same results as MCMC methods. We simulated data from *K* = 5,10 and 20 studies. We used five values of between‐study variance *τ* ^2^ = 0,0.029,0.069,0.206,1.302, because these values correspond to *I* ^2^ = 0*%*,30*%*,50*%*,75*%*,95*%* 25. Additional details of the simulation study are provided in the Supporting Information (S4).

Here, we report the results of the simulation study to compare methods of random‐effects meta‐analysis.

### 5.1 Comparing frequentist properties of Bayesian methods and DL

**Figure 1:**
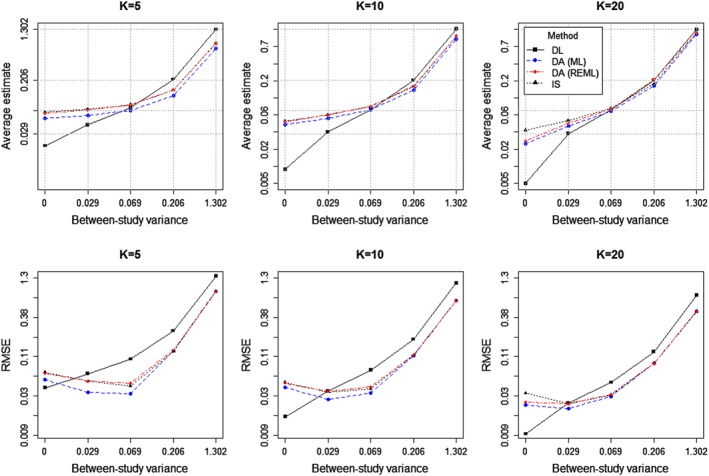
Average estimates [top row] and root mean squared error (RMSE) [bottom row] of between‐study variance *τ* ^2^ from the simulation study, using 20 000 simulations for each combination of *τ* ^2^ and *K*, plotted on the logarithm scale. In each case, DL denotes conventional estimation by the DerSimonian and Laird procedure. DA and IS denote Bayesian methods by data augmentation and importance sampling, respectively.

**Figure 2:**
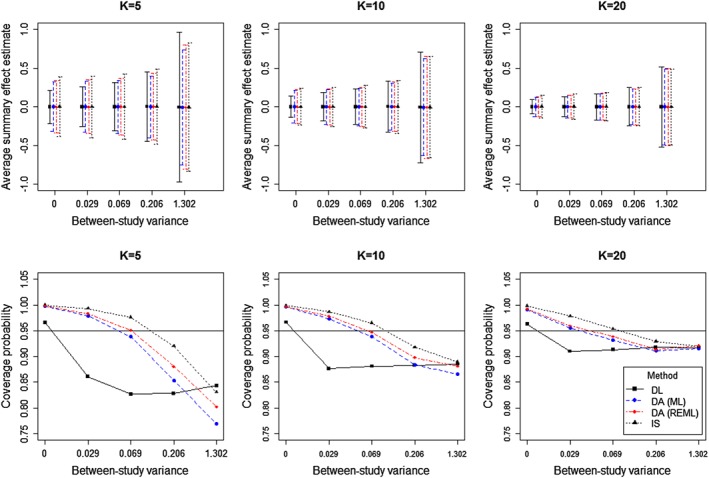
Average estimates with corresponding 95*%* intervals [top row] and coverage probabilities of estimated 95*%* intervals for the summary effect *μ* [bottom row] from the simulation study, using 20 000 simulations for each combination of *τ* ^2^ and *K*, plotted on the logarithm scale. In each case, DL denotes conventional estimation by the DerSimonian and Laird procedure. DA and IS denote Bayesian methods by data augmentation and importance sampling, respectively.

Figure 1 summarises the average estimators of between‐study variance *τ* ^2^ from the simulation studies using 20 000 meta‐analyses simulated under each combination of *τ* ^2^ and *K*. When *τ* ^2^ = 0, the results demonstrate that the DL procedure slightly overestimates *τ* ^2^, even though the data are homogeneous. The two Bayesian methods, incorporating information on heterogeneity, behave similarly in comparison with the conventional method by DL estimation. Specifically, when the DL estimator of *τ* ^2^ is extremely low, both Bayesian methods allow appropriately for imprecision in *τ* ^2^; yielding a larger estimate of *τ* ^2^ and providing a wider interval for the summary intervention effect *μ* on average (Figure 2). On the contrary, methods for Bayesian meta‐analysis provide a reduced estimate for *τ* ^2^ and a narrower interval for *μ* on average, when the DL estimator of *τ* ^2^ is high. As the number of studies *K* increases, average estimates of *τ* ^2^ follow a similar pattern, but the discrepancies between methods fade for a given $\tau^{2}\geq 0.029$.

Figure 1 displays the root mean squared error (RMSE) of the different estimators for *τ* ^2^. When *τ* ^2^ = 0, the Bayesian methods implementing an inverse‐gamma(1.14,0.08) prior for *τ* ^2^ yield larger RMSE than the frequentist approach using the DL procedure, but perform better than the DL procedure when *τ* ^2^ is sufficiently large ( $\tau^{2}\geq 0.029$).

For each method, we display coverage probabilities and average lengths of 95*%* intervals for *μ* graphically in Figure 2. For importance sampling and data augmentation, it is important to note that these results are not based on the credible intervals for *μ*, but have been obtained from the point estimates and model‐based standard errors for *μ*. Although we expect the model‐based credible intervals to be typically wider and have higher coverage probabilities than reported here, the intervals provided are useful to compare methods because they would be similarly influenced by *τ* ^2^. Coverage probabilities are the proportion of nominal estimated 95*%* intervals that cover the true value *μ* = 0, using the standard normal quantile. When *τ* ^2^ = 0, the coverage probabilities for all methods are greater than 95*%* because each Bayesian method provides an increased estimate for *τ* ^2^, and the DL procedure can overestimate *τ* ^2^. In general, the coverage probabilities decrease and the interval lengths widen with increasing *τ* ^2^. For non‐zero *τ* ^2^ values, these results corroborate findings of other simulation studies 26, 27, which demonstrate severe under‐coverage of Wald intervals for *μ* based on DL estimation. Under most scenarios, Bayesian methods outperform conventional DL estimation, providing higher coverage probabilities that are typically closer to 0.95.

### 5.2 Comparing Bayesian methods using data augmentation and importance sampling

Comparing methods to implement a prior for the between‐study variance, it appears that the method using importance sampling generally gives a slightly higher estimate for *τ* ^2^, on average, than the method by ML estimation via data augmentation. Nonetheless, the method by data augmentation performs approximately as well as the method by importance sampling when $\tau^{2}\geq 0.029$, as the number of studies *K* in the meta‐analysis increases. The results show that estimators of *τ* ^2^ obtained through data augmentation by REML estimation are, on average, very close to the importance weighted estimators of *τ* ^2^, regardless of the number of studies *K* in the meta‐analysis.

We compare the estimated coverage probabilities of intervals for *μ* between the two Bayesian approaches for meta‐analysis. As shown in Figure 2, for *K* = 5, the intervals from importance sampling tend to be wider and provide higher coverage than those estimated using data augmentation. For *τ* ^2^ = 0.069, the coverage of the intervals from importance sampling is greater than 0.95 whereas data augmentation has lower than nominal coverage. When *K* = 10 and 20, the coverage and lengths of intervals for *μ* follow a similar pattern, but results from the three different methods of analysis are closer as the number of studies in the meta‐analysis increases. We note that the interval for *μ* obtained through data augmentation is, on average, closer to the interval based on importance sampling, if REML estimation is used rather than ML estimation. Overall, the results from our simulation studies are reassuring and demonstrate that meta‐analysis results from the proposed Bayesian approach by data augmentation perform similarly to those from the existing method for a fully Bayesian meta‐analysis by importance sampling with increasing *τ* ^2^.

### 5.3 Comparing methods using Markov chain Monte Carlo and importance sampling

The main results of our analyses are shown in Table 3; the same data were used for both methods of Bayesian meta‐analysis implementing an inverse‐gamma(1.14,0.08) prior for *τ* ^2^. Table 3 shows estimates relating to the summary intervention effect *μ* and the between‐study variance *τ* ^2^. The results from performing Bayesian meta‐analysis using importance sampling and MCMC methods are almost identical, as we expect. Discrepancies between importance weighted estimators for *μ* and *τ* ^2^ and estimates from MCMC are only apparent in meta‐analyses simulated with the highest heterogeneity value *τ* ^2^ = 1.302. There is such a high amount of variation under this scenario, and we would require many more simulations over 1000 for this extreme test of agreement between the two methods.

**Table 3:** Properties of estimates for *μ* and *τ* ^2^ from the simulation study with *K* = 5 studies, using 1000 simulations for each *τ* ^2^ value. In each case, IS denotes values using Bayesian methods by importance sampling. ‘Z length’ denotes the average length of the nominal 95*%* interval for *μ* using the standard normal quantile, and ‘Z coverage’ denotes the proportion of nominal 95*%* intervals that cover the true value, using the standard normal quantile.

**Properties of estimates for *μ***								
	**Empirical mean**		**Empirical std dev**		**Z length**		**Z coverage**	
	IS	MCMC	IS	MCMC	IS	MCMC	IS	MCMC
*τ* ^2^ = 0	− 0.001	− 0.001	0.106	0.106	0.771	0.771	0.9993	1
*τ* ^2^ = 0.029	0.003	0.003	0.143	0.144	0.800	0.805	0.995	0.995
*τ* ^2^ = 0.069	0.001	0.001	0.170	0.169	0.840	0.842	0.985	0.985
*τ* ^2^ = 0.206	0.005	0.005	0.260	0.260	0.971	0.984	0.996	0.996
*τ* ^2^ = 1.302	0.013	0.012	0.556	0.554	1.654	1.839	0.991	0.992
**Properties of estimates for *τ*^2^**								
	**Empirical mean**		**Empirical std dev**	
	IS	MCMC	IS	MCMC				
*τ* ^2^ = 0	0.064	0.064	0.018	0.018				
*τ* ^2^ = 0.029	0.073	0.073	0.027	0.027				
*τ* ^2^ = 0.069	0.084	0.084	0.040	0.040				
*τ* ^2^ = 0.206	0.141	0.142	0.112	0.111				
*τ* ^2^ = 1.302	0.767	0.787	0.646	0.682				

MCMC, Markov chain Monte Carlo.

## 6 Extensions to more complex meta‐analysis models

In principle, informative prior distributions for between‐study variance could be used in more complex meta‐analysis models. In a multivariate meta‐analysis combining estimates of intervention effect for multiple outcomes over multiple studies, the priors could be applied directly if the heterogeneity variances and correlations are separated in the between‐study variance‐covariance matrix 28, 29. In a meta‐regression assessing the relationship between one or more study‐level covariates and intervention effect, informative prior distributions derived for heterogeneity in a standard random‐effects meta‐analysis can be used, as demonstrated by Jackson *et al.* 30.

Obtaining empirical evidence on heterogeneity for specific meta‐regression models would be very difficult, because there are many combinations of study‐level covariates that could be used in the regression models. Because the covariates included in a meta‐regression model might be expected to explain some heterogeneity, we anticipate the between‐study variance to be smaller than in the corresponding random‐effects meta‐analysis. If the between‐study variance is indeed smaller in the meta‐regression, using priors derived in the context of meta‐analysis for meta‐regression models can be viewed as being conservative.

Network meta‐analyses comparing multiple interventions are becoming increasingly widely used. The method involves the simultaneous analysis of both direct comparisons within studies and indirect comparisons across studies, via a common comparator. Over recent years, a variety of approaches to modelling the variance parameters in a network meta‐analysis have been proposed 28, 31, 32, 33. Models for network meta‐analysis can be expressed as standard uni‐variate meta‐regression models when all studies are two‐arm trials 34. Because the methods developed in this paper are applicable to meta‐regression, this means that our methods are relevant and directly applicable to network meta‐analysis of two‐arm trials.

Suppose a simple network compares *p* + 1 different interventions (0,…,*p*). We regard intervention 0 as the overall reference intervention in the network, and the *p* contrasts with the reference intervention, *μ*_01_,*μ*_02_,…,*μ*_0*p*_, are referred to as the basic parameters in the network meta‐analysis. Under the assumption of consistency, the remaining intervention contrasts can be written in terms of the basic parameters, for example *μ*_12_ = *μ*_02_ − *μ*_01_ 35.

For an approximate Bayesian network meta‐analysis using data augmentation, we assume the same heterogeneity variance *τ* ^2^ for each intervention contrast in order to use standard meta‐regression techniques 36. We follow the method proposed in Section 2.1.1 for a standard pairwise meta‐analysis, fitting the following random‐effects meta‐regression model with no intercept term:

(3) $$Y_{i}∼N\left(\sum_{j}\mu_{0j}I_{ij},\sigma_{i}^{2}+\tau^{2}\right),$$

where *Y*_*i*_ is the intervention effect from the *i*‐th study (*i* = 1,…,*K* + *K*0). *I*_*ij*_ is a dummy variable corresponding to basic parameter *μ*_0*j*_ in the network meta‐analysis for *j* = 1,…,*p*. If study *i* includes the reference intervention 0 for the whole network, then *I*_*ij*_ = 1 for the other intervention *j* in that study and *I*_*ij*_ = 0 otherwise. If study *i* does not include the reference intervention, then *I*_*ij*_ = 1 if intervention *j* is the non‐reference arm in the study, *I*_*ij*_ =− 1 if intervention j is the reference arm in the study and *I*_*ij*_ = 0 otherwise. In each of the *K*_0_ artificial studies representing the prior for *τ* ^2^, we set *I*_*ij*_ to be zero for all *j* . That way, the effects of the artificial studies are centred at zero, as in the standard pairwise meta‐analysis setting, and each *μ*_0*j*_ estimates the combined effect of intervention *j* relative to intervention 0 for the observed studies.

### 6.1 Application to an example network meta‐analysis

As an example application to network meta‐analysis, we use a data set comprising 24 studies comparing four interventions for smoking cessation: *A* = no intervention, *B* = self‐help, *C* = individual counselling, *D* = group counselling. This data set has been analysed previously by Hasselblad 37 and Lu and Ades 38 among others. The outcome is successful smoking cessation at 6–12 months. There is direct evidence available on all six possible pair‐wise comparisons: AB (3 studies), AC (15 studies), AD (2 studies), BC (2 studies), BD (2 studies), CD (4 studies). Two of the studies are three‐arm studies, which we treat as independent two‐arm studies, in order to use standard methods for meta‐regression.

As an informative prior for the between‐study variance *τ* ^2^, we chose an inverse‐gamma(1.39,0.13) distribution. This prior corresponds to the predictive distribution for between‐study variance in a non‐pharmacological meta‐analysis examining a subjective outcome (Table 1).

In a conventional random‐effects meta‐regression using the DL procedure, the estimate of between‐study variance is quite high at 0.60 (95*%* CI: 0.21 to 1.32 obtained iteratively using the Q‐profile method 23). When implementing a Bayesian meta‐analysis with an informative inverse‐gamma prior for *τ* ^2^ using standard MCMC methods, the central estimate (posterior median) for *τ* ^2^ reduces to 0.37, with 95*%* credible interval 0.18 to 0.83 (Table 4). The 95*%* intervals for the log odds ratios *μ*_*AB*_,*μ*_*AC*_ and *μ*_*AD*_ have narrowed somewhat.

**Table 4:** Results from re‐analysing data from the network meta‐analysis to compare interventions for smoking cessation. Bayesian approaches apply an empirically based inverse‐gamma(1.39, 0.13) prior for *τ* ^2^ in a non‐pharmacological meta‐analysis with a subjective outcome.

**Method**	*μ*_*AB*_	(95*%* CI)	*μ*_*AC*_	(95*%* CI)	*μ*_*AD*_	(95*%* CI)	*τ* ^2^	(95*%* CI)
Conventional (DL estimation)	0.46	( − 0.24, 1.16)	0.70	(0.27, 1.12)	0.98	(0.13, 1.84)	0.60	(0.21, 1.32)^1^
Data augmentation by DL	0.35	( − 0.03, 0.72)	0.59	(0.38, 0.80)	0.85	(0.34, 1.37)	0.09	(0.19, 1.07)^1^
Data augmentation by ML	0.42	( − 0.13, 0.96)	0.64	(0.32, 0.97)	0.92	(0.23, 1.61)	0.31	(0.19, 1.07)^1^
Data augmentation by REML	0.42	( − 0.14, 0.99)	0.65	(0.31, 1.00)	0.93	(0.22, 1.64)	0.35	(0.19, 1.07)^1^
MCMC	0.43	( − 0.16, 1.05)^2^	0.66	(0.31, 1.04)^2^	0.94	(0.20, 1.70)^2^	0.37	(0.18, 0.83)^2^

1 The confidence interval for *τ* ^2^ is obtained iteratively via the Q‐profile method 23.

2 Posterior medians and 95*%* credible intervals are reported for the log odds ratios *μ*_*AB*_,*μ*_*AC*_ and *μ*_*AD*_ and for the common heterogeneity variance *τ* ^2^.

DL, DerSimonian and Laird; ML, maximum likelihood; REML, restricted maximum likelihood; MCMC, Markov chain Monte Carlo;

Results from approximate Bayesian analyses using data augmentation compare favourably to those from fully Bayesian analyses using MCMC methods (Table 4). In particular, point estimates and corresponding credible intervals for the log odds ratios *μ*_*AB*_,*μ*_*AC*_ and *μ*_*AD*_ obtained using REML estimation by data augmentation are close to those obtained using standard MCMC methods. As before, we find that discrepancies between results from data augmentation and MCMC methods are most apparent when the data augmentation method is implemented by the DL procedure.

## 7 Discussion

Numerous meta‐analyses in healthcare research combine results from only a small number of studies, for which estimation of between‐study heterogeneity is difficult. Bayesian meta‐analysis is advantageous because it allows the incorporation of external information on heterogeneity between studies to potentially improve precision of results. In this paper, we have described an easy‐to‐use method for implementing Bayesian meta‐analysis, based on commonly used frequentist methods via data augmentation. We have demonstrated the use of this method and provided examples where point estimates obtained through data augmentation are similar to more computationally intensive approaches using MCMC methods and importance sampling techniques.

Our method for an approximate Bayesian meta‐analysis with a inverse‐gamma prior for the between‐study variance *τ* ^2^ was developed by working with the logarithm of the between‐study precision 1/*τ* ^2^, and matching the corresponding likelihood from some pseudo data to the Gamma prior for 1/*τ* ^2^ in terms of  − 2log(*τ*). The more straightforward approach would have been to work with the untransformed between‐study precision 1/*τ* ^2^ and match the likelihood from some pseudo data to the Gamma prior for 1/*τ* ^2^. In fact, our initial method to an approximate Bayesian meta‐analysis followed this approach, using *K*_0_ = 2(*α* − 1) studies with effects $y_{0}=\sqrt{2\beta /K_{0}}$ to represent an inverse‐gamma(*α*,*β*) prior for the between‐study variance *τ* ^2^. But, it was immediately obvious through applications to examples that this approach does not work well and is not recommended.

Bayesian analysis via data augmentation has already been proposed by Sander Greenland, for example in papers that describe methods for regression analysis 11, 12, 14. To our knowledge, this paper represents the first application of data augmentation as a method for applying a prior to a variance parameter and for Bayesian meta‐analysis. Compared with alternative methods by importance sampling and MCMC, data augmentation is the most computationally rapid, running in about the same time as maximum likelihood estimation. Our approach by data augmentation does not require convergence checking and is simple to use with the *R* or *Stata* computing code available in the Supporting Information (S2.1). All that is required of the analyst is to input their observed study data and selected inverse‐gamma prior parameters in order to implement the Bayesian meta‐analysis with an inverse‐gamma prior for the between‐study variance *τ* ^2^. The representation of the prior as pseudo data in data augmentation provides an insight into the strength of the prior and the information that the prior is incorporating into the Bayesian analysis.

*Review Manager* (*RevMan*) is the Cochrane Collaboration's software for preparing and maintaining Cochrane reviews 39. *RevMan* assists in the preparation of protocols and full reviews and can perform meta‐analysis of the study data entered. Compared with alternative approaches for Bayesian meta‐analysis, the proposed method by data augmentation has the advantage that it could potentially be made accessible in user‐friendly software such as *RevMan*, once meta‐regression techniques are made available. Should an analyst prefer to use standard approaches by MCMC, alternative methods are nevertheless useful. For example, importance sampling techniques provide a useful diagnostic check that MCMC estimates are correct.

In a simulation study, we have formally compared the proposed method for an approximate Bayesian meta‐analysis using data augmentation to a fully Bayesian meta‐analysis using importance sampling techniques. When conventional DL procedures produce an extremely low estimate of between‐study variance *τ* ^2^, the Bayesian approaches tend to be more conservative, yielding a larger *τ* ^2^ estimate and a wider interval for the summary effect *μ*. When the conventional heterogeneity estimate is high, the two methods typically provide a reduced estimate for *τ* ^2^ and a more precise estimate for *μ*. We would not expect methods by data augmentation and importance sampling to lead to precisely the same results because data augmentation is approximately Bayesian and also importance sampling is susceptible to simulation error. Nonetheless, our simulation study has shown good agreement between point estimates from the two methods, particularly as the number of studies increases. Results from the simulation study demonstrate that meta‐analysis results obtained through data augmentation are, on average, closer to those resulting from importance sampling, when implemented using REML estimation rather than DL or ML estimation. The DL procedure had overall poorer performance characteristics. These findings are not surprising because REML estimation has a Bayesian justification 40, and the DL procedure has potential for bias in the method‐of‐moments estimator of the between‐study variance *τ* ^2^ that uses estimates of study‐specific variances 41, 42.

The simulation studies we have conducted to compare methods by data augmentation and importance sampling are useful to help understand the validity of data augmentation as an alternative to MCMC. We showed in a further simulation study that importance sampling yields approximately identical results to standard MCMC methods within *WinBUGS*. Point estimates obtained through data augmentation and importance sampling have shown close similarities, but further work needs to be done in order to obtain comparable credible intervals for *τ* ^2^. In data augmentation, the current method is to obtain an interval iteratively, as for ML estimation, via the Q‐profile method. Based on previous research, underlying values of heterogeneity variance conform better to a log‐normal or log *t*_5_ distribution than an inverse‐gamma distribution 5, 6. Limitations of our method by data augmentation are that we are restricted to use of an inverse‐gamma prior for *τ* ^2^ and that we have not estimated posterior percentiles. For these purposes, numerical integration routines can be used, as can importance sampling techniques which are limited in accuracy only by the length of time required to draw sufficient samples from the posterior distribution 7.

A disadvantage of the easy‐to‐use methods by data augmentation, numerical integration and importance sampling is that they use normal approximations for the observed study‐level effects. When modelling the *CDSR* data set to obtain predictive distributions for the between‐study variance, we used the binomial likelihood approach to analyse binary outcome data, which is preferable in principle 41. The approximate method can be expected to provide biased estimates, particularly in cases where studies are small or the event is rare. More research is needed in order to extend the methods described in this paper to exact likelihood approaches based on the binomial within‐study distribution. This would be a challenging area for further work.

We have demonstrated the use of data augmentation for implementing a conjugate prior for the residual between‐study variance in a network meta‐analysis of two‐arm trials. For simplicity, our approach to network meta‐analysis makes an assumption of ‘consistency’, which is reasonable for the purpose of our example, but more sophisticated methods are available 33. When combining the results of direct and indirect comparisons in practice, it is important to examine the extent to which these results are consistent (in agreement) with each other. Our simple approach to network meta‐analysis assumed that all heterogeneity variances in the network were equal, which will be adequate in some network meta‐analyses. It would be much more difficult to specify informative priors, when allowing heterogeneity variances to differ across some treatment comparisons, because the priors for heterogeneity must ensure a valid between‐study variance‐covariance matrix 28. We plan to extend our work in the future to explore how to specify informative priors for heterogeneity while allowing the heterogeneity variances to differ across intervention comparisons. 

In summary, Bayesian meta‐analysis with an informative prior for the between‐study variance is recommended for use in small meta‐analyses. Bayesian estimation need not be computationally difficult and require specialist software, but can be implemented using our easy‐to‐use method by data augmentation. We hope that the work developed in this paper could help increase the accessibility of Bayesian meta‐analysis and promote Bayesian meta‐analysis for use in many applications.

## Supporting information



Supporting info item
