# Supplementary material for: Implementing informative priors for heterogeneity in meta‐analysis using meta‐regression and pseudo data
Source: Stat Med. 2016 Aug 30;35(29):5495–511. doi: 10.1002/sim.7090 (PMC5111594; doi:10.1002/sim.7090)
Supplement: Supplementary file 1 — Supporting info item [file SIM-35-5495-s001.pdf]

Supporting Information

S1 Visual representation of pseudo data representing priors for between-study variance τ^2

We present three sets of artificial studies, which may serve as the three different inverse-gamma distributions for τ^2 displayed in Figure S1(a). The intervention effect is highest in magnitude for the studies representing the inverse-gamma(1,1) distribution with the greatest prior mean for τ^2 (Figure S1(b)). The inverse-gamma(1,1) distribution in Figure S1(a) has the greatest variance and is represented by just two studies in Figure S1(b), compared with four studies for the inverse-gamma(2,1) prior and six studies for the inverse-gamma(3,0.5) prior with the greatest precision. The intervention effect is lowest in magnitude for the studies representing the inverse-gamma(3,0.5) prior with the lowest mean.

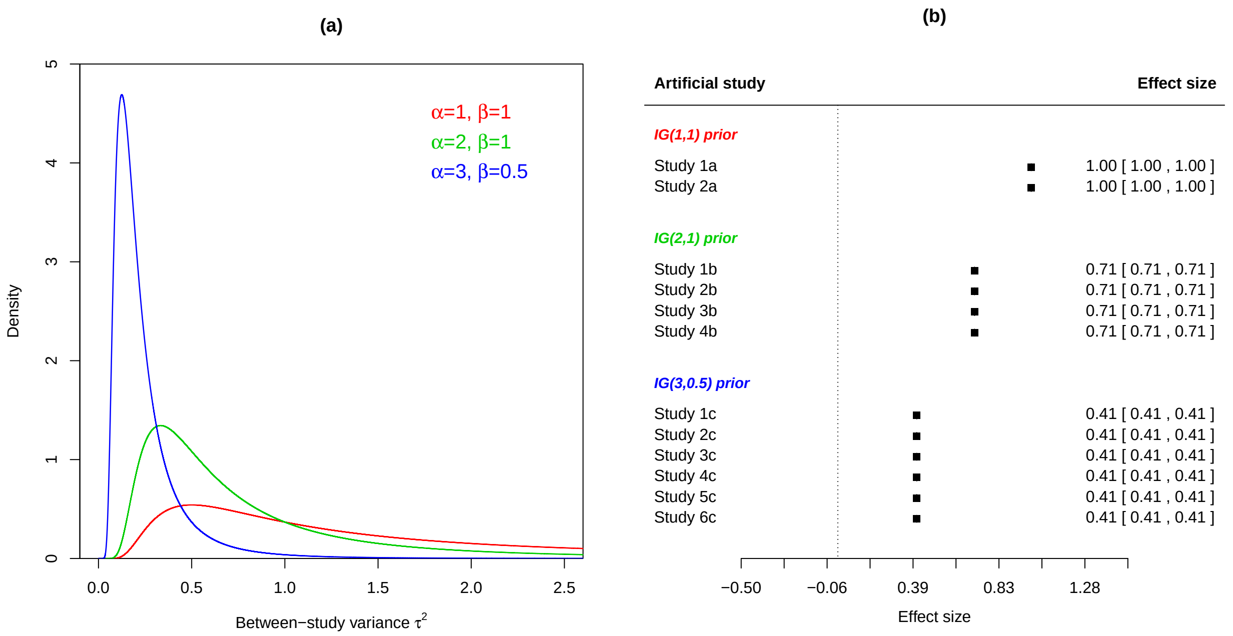

Figure S1: (a) Inverse-gamma(α, β) prior distributions for τ^2. (b) Sets of pseudo studies to represent the inverse-gamma(α, β) priors for τ^2.

S2 Computing code

S2.1 Approximate Bayesian meta-analysis using meta-regression and pseudo data

S2.1.1 *R* code

This *R* code produces the results from an approximate Bayesian meta-analysis using data augmentation by maximum likelihood estimation, as described in Section 2.1. An inverse-gamma prior is declared for the between-study variance τ^2. The *metafor* package [1] must be installed for this code to work.

The function "dataaugmentation.rma" is called to produce the results, where dataaugmentation.rma is a function of four arguments. These four arguments are:

y: vector of length K with the observed intervention effects (in our case, log odds ratios).
v: vector of length K with the corresponding within-study variances.
shape: the shape parameter of the inverse-gamma prior for τ^2 (default is 1.14).
scale: the scale parameter of the inverse-gamma prior for τ^2 (default is 0.08).

The function returns a list containing the following components:

mu: the estimated summary intervention effect.
mu.int: the corresponding 95% credible interval for the estimated summary intervention effect.
tau2: the estimated between-study variance.
tau2.int: the corresponding 95% credible interval for the estimated between-study variance.

```
dataaugmentation.rma<-function(y,v,shape=1.14,scale=0.08){

        K<-length(y)

#  Set up "prior" studies
        K0<-round(2*shape)
        y.prior<-rep(sqrt(2*scale/K0),K0)
        v.prior=rep(1/10^20,K0)

#Place observed and unobserved data in separate strata
        Y<-c(y,y.prior)
        Sigma2<-c(v,v.prior)
        type<-c(rep(1,length(y)),rep(0,length(v.prior)))

# Perform Bayesian meta-analysis using meta-regression by REML estimation
        RE.MA<-rma(Y, Sigma2,mods=~type-1,method="REML")
        mu<-RE.MA$b[,1]
        mu.int<-c(min(RE.MA$ci.lb,RE.MA$ci.ub),max(RE.MA$ci.lb,RE.MA$ci.ub))
        tau2<-RE.MA$tau2
        tau2.int<-c(min(confint.rma.uni(RE.MA)$random[,2][1],
        confint.rma.uni(RE.MA)$random[,3][1]),
        max(confint.rma.uni(RE.MA)$random[,2][1],
        confint.rma.uni(RE.MA)$random[,3][1]))

        output<-list(mu,mu.int,tau2,tau2.int)
        names(output)<-c("mu","mu.int","tau2","tau2.int")
```

```
        class(output)<-"dataaugmentation.rma"
        return(output)     }
```

Running the code below in *R* [2] produces the results in Table 2 of Section 4 for re-analysing study data from the published meta-analysis evaluating the effectiveness of fluoride for lower limb pain (Example 1), using REML estimation by data augmentation.

```
dataaugmentation.rma(y=c(0.653926467406664, -0.287682072451781, 4.1461377316513,
2.40694510831829),v=c(0.203879598662207, 0.333333333333333, 2.09967998456912,
0.253068693693694),shape=1.45,scale=0.18)
```

S2.1.2 *Stata* code

For meta-analysts who prefer to use *Stata* [3], the code to follow will yield equivalent results. This code makes use of the *metareg* command [4].

```
// declare an informative inverse-gamma(1.45,0.18) prior for the between-study variance tau2

*set mean and std dev of prior for 1/tau2
local shape 1.45
local scale 0.18

*derive the number of artificial studies and their effect
local K=round(2*‘shape’)
confirm integer number ‘K’

local y=sqrt(2*‘scale’/‘K’)

*input meta-analysis data, plue an additional row for the prior
clear
input type y v

1 0.653926467406664 0.203879598662207
1 -0.287682072451781 0.333333333333333
1 4.1461377316513 2.09967998456912
1 2.40694510831829 0.253068693693694
0 . .
end

*set up the artificial studies
replace y=‘y’ if type==0
replace v=1E-20 if type ==0
expand ‘K’ if type==0
gen s=sqrt(v)


*approximate Bayesian analysis
metareg y type, wsse(s) reml z noconst
```

S2.2 *WinBUGS* code for implementing MCMC

The *WinBUGS* [5] code provided below produces the results in Table 2 of Section 4 for re-analysing study data from Example 1 using *MCMC*.

```
model{
        for(i in 1:K){
                prec.y[i]<-1/v[i]
                y[i]~dnorm(theta[i],prec.y[i])
                theta[i]~dnorm(mu,invtausq)
                }

                mu~dnorm(0,0.000001)
                invtausq~dgamma(shape,scale)
                tausq<-1/invtausq
        }

#Data for example 1
list(y=c(0.653926467406664, -0.287682072451781, 4.1461377316513, 2.40694510831829
),v=c(0.203879598662207, 0.333333333333333, 2.09967998456912, 0.253068693693694
),K=4,shape=1.45,scale=0.18)
```

S2.3 *R* code for implementing importance sampling

The *R* code to follow is a modified version of the code provided by by Turner *et al* [6]. The code defines a *R* function "importance.rma" for performing a fully Bayesian meta-analysis with an inverse-gamma prior for the between-study variance τ^2, using importance sampling techniques. The code makes use of the *Hmisc* package [7], so this should be downloaded first. Also required is a function that we have called "imp.weights" , in order to compute the importance weights $w(\mu, \tau^2)$.

```
importance.rma<-function(y,v,sims,seed=1,lower=0.025,upper=0.975,scale=4,
               shape_prior,scale_prior){

        set.seed(seed)

#Compute parameters for the simple distribution that we want to sample from
 #----------------------------------------------------------------------------
        tau2_p<-1/(shape_prior/scale_prior)
        re_ws<-1/(v+tau2_p)
        v_p2<-scale/sum(re_ws)
        mu_p<-sum(re_ws*y)/sum(re_ws)

#Draw mu and tau2 from the simpler distribution
#--------------------------------------------
        mus<-rnorm(sims,mu_p,v_p2^0.5)
        tau2s<-1/(rgamma(sims,shape_prior,scale_prior))
        sims_mat<-matrix(nrow=sims,ncol=2) #Store simulations in a matrix
                sims_mat[,1]<-mus
                sims_mat[,2]<-tau2s

#Weight the simulated observations
#--------------------------------
```

```
weights<-apply(sims_mat,1,FUN=imp_weights,y=y,v=v,mu_p=mu_p,v_p2=v_p2)

den<-sum(weights) #Compute posterior moments for mu, tau2
Emu<-sum(weights*mus)/den
Etau2<-sum(weights*tau2s)/den
Emu2<-sum(weights*mus^2)/den
Etau4<-sum(weights*tau2s^2)/den

sd_mu<-sqrt(Emu2-Emu^2) #Compute std dev for mu, tau2
sd_tau2<-sqrt(Etau4-Etau2^2)

mc_var_mu<-sims*var(weights*mus)/(den^2)
        -2*sum(weights*mus)*sims*cov(weights*mus,weights)/(den^3)
        +((sum(weights*mus))^2)*sims*var(weights)/(den^4)
mc_var_tau2<-sims*var(weights*tau2s)/(den^2)
        -2*sum(weights*tau2s)*sims*cov(weights*tau2s,weights)/(den^3)
        +((sum(weights*tau2s))^2)*sims*var(weights)/(den^4)


pr<-weights/den

mu_q<-as.numeric(wtd.quantile(mus,pr,normwt=TRUE,probs=c(lower,0.5,upper)))
tau2_q<-as.numeric(wtd.quantile(tau2s,pr,normwt=TRUE,probs=c(lower,0.5,upper)))


output<-list(Emu,sqrt(mc_var_mu),sd_mu,mu_q[2],c(mu_q[1],mu_q[3]),
          Etau2,sqrt(mc_var_tau2),sd_tau2,tau2_q[2],c(tau2_q[1],tau2_q[3]))

names(output)<-c("posterior_mean_of_mu","mc_error_mu","sd_mu","median_mu","mu_int",
        "posterior_mean_of_tau2","mc_error_tau2","sd_tau2","median_tau2","tau2_int")

class(output)<-"importance.rma"
return(output)
}

imp_weights<-function(x,y,v,mu_p,v_p2){
        prod(dnorm(y,mean=x[1],sd=(v+x[2])^0.5))/(dnorm(x[1],mu_p,sqrt(v_p2)))
                }
```

We assume that the study data from the meta-analysis are stored as length K vectors of observed intervention effects (in our case, log odds ratios) and corresponding within-study variances. To perform Bayesian meta-analysis with an inverse-gamma(1.45,0.18) prior for τ^2, with a sample of 1000000 simulations, we run the code below in *R*. This code returns the posterior means, medians, standard deviations and 95% credible intervals, along with the MC errors, for the summary intervention effect μ and the between-study variance τ^2.

```
importance.rma(y,v,sims=1000000,shape_prior=1.45,scale_prior=0.18)
```

S3 Models for heterogeneity

We used Bayesian hierarchical models to analyse study data from each binary outcome meta-analysis in the data set, whilst investigating the influence of meta-analysis characteristics on the degree of heterogeneity among results of included studies. Use r_{ij}^{a} to denote the number of events in treatment arm a ($a = C, T$ for Control and Treatment arm being compared) in study i of meta-analysis j, from a total number of n_{ij}^{a} patients, each assumed to have probability π_{ij}^{a} of having the event. Within each meta-analysis j, a random-effects model with binomial within-study likelihoods was fitted to binomial outcome data r_{ij}^{a}/n_{ij}^{a} from each study i ($a = C, T$ for Control and Treatment arm being compared):

$$r_{ij}^{C} \sim \text{Binomial}(\pi_{ij}^{C}, n_{ij}^{C})$$
$$r_{ij}^{T} \sim \text{Binomial}(\pi_{ij}^{T}, n_{ij}^{T}).$$

$$\text{logit}(\pi_{ij}^{C}) = \alpha_{ij} - \theta_{ij}/2$$
$$\text{logit}(\pi_{ij}^{T}) = \alpha_{ij} + \theta_{ij}/2$$

$$\theta_{ij} \sim N(\mu_j, \tau_j^2).$$

where the baseline odds, α_{ij}, are fixed effects, and the treatment effects (log odds ratios θ_{ij} have normal random-effects distributions. In the defined model, μ_j corresponds to the combined intervention effect for meta-analysis j, and τ_j^2 represents the underlying between-study heterogeneity.

Across meta-analyses, regression models were fitted to underlying values of between-study variance τ_j^2, assuming an inverse-gamma distribution. In the defined model, x_{1j}, x_{2j} are indicators for whether the meta-analysis indexed j had an outcome which was all-cause mortality or semi-objective. Likewise, z_{1j}, z_{2j} are binary indicators for whether the intervention comparison was pharmacological vs placebo/control or pharmacological vs pharmacological respectively. β_1 and β_2 are regression coefficients which represent average differences between each outcome type and the reference group of subjective outcomes, whereas the fixed effects γ_1 and γ_2 denote the average differences between each intervention comparison type and the reference group of non-pharmacological intervention comparisons. Additional fixed effects $\delta_1, ..., \delta_{10}$ were added to the regression models to investigate the influence of medical specialities.

$$\tau_j^2 \sim \text{Inverse-gamma}(r_{xz}, \lambda_j)$$

where

$$\log(\lambda_j) = \beta_0 + \beta_1 x_{1j} + \beta_2 x_{2j} + \gamma_1 z_{1j} + \gamma_2 z_{2j} + \delta_1 a_{1j} + ... + \delta_{10} a_{10j}.$$

All models were fitted using MCMC within the *WinBUGS* [5] software and results were based on 50,000 iterations following a burn-in period of 10,000 iterations. This was sufficient to achieve convergence. Convergence diagnostics were run on the 50,000 iterations after burn-in. We monitored convergence using the Brooks-Gelman-Rubin statistic [9], as implemented in *WinBUGS*. For a single MCMC chain, convergence was checked graphically via trace plots and autocorrelation plots. Vague normal(0, 100) prior distributions were assigned to each summary effect μ_j and all regression coefficients, as recommended by Spiegelhalter *et al.* [10]. For each pair-wise combination

of outcome types u and types of intervention comparison v, we assigned the shape parameter r_{xz} an exp(0.001) prior distribution which is slowly decreasing on the positive real line [5]. We carried out sensitivity analyses to investigate how the results depend on the choice of non-informative prior distributions. The estimated parameters for each Bayesian hierarchical model using different priors were very similar.

For each research setting defined by the type of outcome x and type of intervention comparison z, we obtained a predictive distribution for heterogeneity τ^2_{new} in a new meta-analysis j in that setting, within the full Bayesian model. For example the predictive distribution for τ^2 in a new binary outcome meta-analysis assessing a semi-objective outcome ($x_{2new} = 1$), comparing a pharmacological intervention against a placebo ($z_{1new} = 1$) in the reference medical area was obtained by monitoring the following:

$$\begin{aligned} \tau^2_{new} &\sim \text{Inverse-gamma}(r_{21}, \lambda_{new}) \\ \log(\lambda_{new}) &= \beta_0 + \beta_2 + \gamma_1. \end{aligned}$$

In our initial analyses, we compared the fit of various models which differed according to the meta-analysis characteristics that the shape and scale parameters of the inverse-gamma distribution were allowed to depend on. The inclusion of indicators for medical area in the model for $\log(\lambda_j)$ led to improvement in model fit based on deviance information criterion (DIC). However, the obtained predictive distributions tended to be very similar across medical areas. For this reason, we report a set of predictive distributions for τ^2 expected in research settings defined only by outcome type and type of intervention comparison. These distributions were obtained by fixing each indicator for medical area equal to the corresponding proportion of meta-analyses in the data. We consider it undesirable to report more predictive distributions than necessary.

We used *WinBUGS* to obtain 50,000 samples from each posterior distribution of τ^2_{new} after convergence. To allow us to summarize the distributions easily, we report inverse-gamma distributions fitted to each sample of values for τ^2_{new}, using the *R* function *fitdistr* in library *MASS* [11]. The predictive distributions provided are approximations of the predictive inverse-gamma distributions obtained under the full Bayesian model within *WinBUGS*. We are content to use this procedure as a pragmatic way to derive informative prior distributions for the between-study variance τ^2.

S4 Simulation study design

Initially we conducted a simulation study with $K = 5$ studies. This represents the situation commonly found in meta-analysis where there are only a small number of studies [8] and Bayesian methods are particularly beneficial. If instead there were many more studies available, then there would be sufficient data, and conventional estimation of the random-effects model would lead to similar inference as Bayesian estimation. The data were simulated using within-study variances of $\sigma_1^2 = 0.009, \sigma_2^2 = 0.046, \sigma_3^2 = 0.122, \sigma_4^2 = 0.265$ and $\sigma_5^2 = 0.600$ in the same way as Jackson *et al.* [12]. These values correspond to the $0\%, 25\%, 50\%, 75\%$ and 100% quantiles of the scaled and truncated chi-squared distribution used by Brockwell and Gordon [13] for generating within-study variances. In order to cover a wide range of scenarios, we used five values of between-study variance $\tau^2 = 0, 0.029, 0.069, 0.206, 1.302$, since these values correspond to $I^2 = 0\%, 30\%, 50\%, 75\%, 95\%$ [14]. For each of the five values of τ^2, we generated 20,000 data sets with true summary effect $\mu = 0$. A different random seed was used for each value of τ^2.

We carried out additional simulation studies, again using 20,000 simulated data sets for each scenario, where different random seeds were used for each combination of K and τ^2. In these additional studies, values of $K = 10$ and 20 were used. For simplicity, and because we are only interested in the impact of increasing the number of studies on the performance of methods for Bayesian meta-analysis, we used the same within-study variances as for the simulation study with $K = 5$ studies, replicated twice for $K = 10$ and four times for $K = 20$ studies. We also used the same five values of between-study variance τ^2.

The random-effects model was estimated for each simulated meta-analysis using Bayesian methods by data augmentation and importance sampling with an empirically-based inverse-gamma$(1.14, 0.08)$ prior for the between-study variance τ^2. This prior has median 0.09 and 95% range (0.02, 1.79). Importance sampling was recently proposed by Turner *et al.* [6], for the purpose of implementing a log-normal prior for between-study variance τ^2 in a Bayesian meta-analysis. In order to make direct comparison with our method for Bayesian meta-analysis by data augmentation, we adapted the method to incorporate inverse-gamma priors for τ^2. In the main simulation study, it was computationally convenient to use importance sampling techniques as the gold standard approach rather than MCMC methods, since importance sampling can easily be implemented in the same software as the method by data augmentation. Importance sampling has yielded approximately equivalent results to MCMC methods in example applications [6].

In Bayesian meta-analysis by data augmentation, we used $K_0 = 2 \approx 2 \times 1.14$ artificial studies with intervention effects $y_0 = \sqrt{2 \times 0.08/K_0} = 0.28$ to augment the observed study data using meta-regression by ML and REML estimation. It was also desired to compare the Bayesian methods to a commonly used frequentist method. Conventional estimation of the random-effects model was carried out for each simulated meta-analysis using the DL procedure [15]. For each method, 95% Wald-type intervals for estimators of μ were obtained using the model-based standard errors. All necessary simulations were implemented using *R* [2], specifically the *metafor* package [1] was used for DL estimation and Bayesian meta-analysis by data augmentation, and the *Hmisc* package [7] was used for Bayesian meta-analysis by importance sampling (see S2 for details).

We assessed the suitability of our simulation studies as a means to justify use of data augmentation as an alternative to standard MCMC methods. To date, conclusions that importance sampling and MCMC methods lead to approximately equivalent meta-analysis results have been based upon

applications of methods to examples. In order to provide more convincing evidence of the strong agreement between results from importance sampling and MCMC, we re-analysed study data from our simulation study with $K = 5$ studies under a fully Bayesian framework using MCMC within *WinBUGS*. For purpose of this investigation, we used only the first 1000 simulated data sets for each τ^2 value because MCMC is computationally expensive and all that is desired here is to check the agreement of results based on importance sampling and MCMC.

References

[1] Viechtbauer W. Conducting meta-analyses in *R* with the metafor package. *Journal of Statistical Software* 2010; **36(3)**:1-48.

[2] *R* Core Team. *R: A Language and Environment for Statistical Computing.* *R* Foundation for Statistical Computing, Vienna, Austria, 2013.

[3] StataCorp. *Stata Statistical Software: Release 14.* College Station, TX: StataCorp LP, 2015.

[4] Harbord R, Higgins JPT. METAREG: Stata module to perform meta-analysis regression. *Statistical Software Components, Boston College Department of Economics*, 2004.

[5] Lunn DJ, Thomas A, Best N, Spiegelhalter DJ. WinBUGS-A Bayesian modelling framework: concepts, structure, and extensibility. *Stat Comput* 2000; **10**:325-337.

[6] Turner RM, Jackson D, Wei Y, Thompson S, Higgins JPT. Predictive distributions for between-study heterogeneity and simple methods for their application in Bayesian meta-analysis. *Statistics in Medicine* 2015; **34(6)**:984-998.

[7] Harrell FE. *Hmisc: Harrell Miscellaneous.* *R* package version 3.10-1, 2012.

[8] Davey J, Turner RM and Clarke MJ, Higgins JPT. Characteristics of meta-analyses and their component studies in the Cochrane Database of Systematic Reviews: a cross-subsectional, descriptive analysis. *BMC Med Res Methodo* 2011; **11**:160.

[9] Brooks SP, Gelman A. General methods for monitoring convergence of iterative simulations. *Journal of Computational and Graphical Statistics* 1998; **7**:434-455.

[10] Spiegelhalter DJ, Abrams KR, Myles JP. *Bayesian Approaches to Clinical Trials and Health-Care Evaluation* (1st edition). Wiley, England, 2004.

[11] Venables WN, Ripley BD. *Modern Applied Statistics with S* (fourth edition). Springer, New York, 2002.

[12] Jackson D. Confidence intervals for the between-study variance in random-effects meta-analysis using generalised Cochran heterogeneity statistics. *Research Synthesis Methods* 2013; **4**: 220229.

[13] Brockwell SE, Gordon IR. A simple method for inference on an overall effect in meta-analysis. *Statistics in Medicine* 2007; 26:4531-4543.

[14] Higgins JPT, Thompson SG. Quantifying heterogeneity in a meta-analysis. *Statistics in Medicine* 2002; **21**:1539-1558.

[15] DerSimonian R, Laird N. Meta-analysis in clinical trials. *Controlled Clinical Trials* 1986; **7**:177-188.
